# Differences between Maximum Tongue Force in Women Suffering from Chronic and Asymptomatic Temporomandibular Disorders—An Observational Study

**DOI:** 10.3390/life13010229

**Published:** 2023-01-13

**Authors:** Marta Carlota Diaz-Saez, Hector Beltran-Alacreu, Javier Gil-Castillo, Alfonso Gil-Martínez

**Affiliations:** 1 Physiotherapy Department, Centro Superior de Estudios Universitarios La Salle, Universidad Autónoma de Madrid, 28023 Madrid, Spain; 2 CranioSPain Research Group, Centro Superior de Estudios Universitarios La Salle, Universidad Autónoma de Madrid, 28023 Madrid, Spain; 3 Programa de Doctorado en Medicina y Cirugía, Universidad Autónoma de Madrid, 28029 Madrid, Spain; 4 Toledo Physiotherapy Research Group (GIFTO), Faculty of Physiotherapy and Nursing of Toledo, Universidad de Castilla-La Mancha, 45071 Toledo, Spain; 5 Neural Rehabilitation Group, Cajal Institute, Spanish National Research Council (CSIC), Av. Doctor Arce, 37, 28002 Madrid, Spain; 6 Unit of Physiotherapy, Hospital La Paz Institute for Health Research (IdiPAZ), 28046 Madrid, Spain

**Keywords:** temporomandibular disorder, pain, tongue, maximum force, fatigue, physiotherapy, rehabilitation

## Abstract

Background: Temporomandibular disorders are craniofacial disorders characterized by the presence of chronic pain in masticatory muscles, with higher incidence in the women population. There is little research that has studied tongue force related to temporomandibular disorders, but there are a lot of studies that have demonstrated the impact of tongue force in vital functions, such as chewing, swallowing, phonation, or breathing. According to this, the aim of this study was to compare the maximum force of the tongue between females with chronic temporomandibular disorders and asymptomatic females. We also wanted to establish whether any relationship existed between the pain and fatigue versus the maximum force developed in females with chronic temporomandibular disorders. Material and methods: A cross-sectional study of 67 women between the ages of 18 and 65 years old was performed. The included women were assigned to one of two groups, according to whether they had chronic temporomandibular disorders or not. The procedure was the same for both groups. Outcome measures included the maximum tongue force, intensity of perceived orofacial pain, and intensity of perceived orofacial fatigue. Results: The results showed significant statistical differences for the maximum tongue force measurement between the chronic temporomandibular disorders group and the control group (*p* < 0.05) for all the movements, except the lip pressure measurement. Furthermore, the analysis revealed significant statistical differences between the intensity of perceived orofacial fatigue between the groups (*p* < 0.05). Moreover, the data showed no significant correlations between variables. Conclusion: The study found significant differences in maximum tongue force when comparing women with chronic temporomandibular disorders and asymptomatic women (being superior in these). Likewise, we found that the intensity of perceived orofacial fatigue after tongue exercises showed significant differences between groups. However, this study reveals no correlations between the intensity of perceived orofacial pain and fatigue and the maximum tongue force.

## 1. Introduction

Temporomandibular disorders (TMDs) are relatively common disorders classified among craniofacial disorders. Some 10% of the population over 18 years of age are affected by TMDs [1], with a higher incidence of symptoms in women aged 25 and older than in men [2]. As indicated by a 2018 systematic review and meta-analysis, women have more than twice the risk of men of suffering from TMDs due to differences in hormonal, cultural, social, occupational stress, pain sensitivity, and health-seeking behaviors [3]. Moreover, women are reported to have more severe pain and greater pressure sensitivity [4]. The most useful diagnostic classification for clinical practice is the Diagnostic Criteria for Temporomandibular Disorders (DC/TMD), which is divided into two axes: physical assessment and the psychosocial state and pain-related disability evaluation [5].

TMDs currently have a non-defined multifactorial etiology [1,2,4]. The primary symptom is the presence of chronic pain in the masticatory muscles, which can refer pain to the neck, the cranium, and the ears [2]. Chronic pain is defined by the International Association for the Study of Pain as a type of pain whose symptoms last continuously longer than 3 months or intermittently for more than 6 months [6]. Due to this chronicity, psychosocial components might play an important role in the onset and persistence of these disorders in both sexes equally [4]. Some studies have observed that patients with depression and catastrophic pain are more likely to develop a TMD [7]. Possible aetiologies of TMD are chronic pathologies of the temporomandibular tissues, such as chondromatosis [8]. Moreover, given that it occurs in other persistent pain procedures, patients with TMDs could initiate compatibles signs and symptoms with peripheral or central sensitization [9]. Likewise, some authors describe a central sensitization event linked to a significant decrease in the pain threshold and a perpetuation of chronic pain [10,11,12]. Central sensitization is maintained by an uninhibited phenomenon of the inhibitory descendent system at various levels, implying that neurons of the dorsal horn become more sensitive to activation from excitatory factors [13,14]. Thilander et al. found that the increase in sensitivity in the temporal and masseter muscles was another important symptom in patients with TMD that added to chronic pain [15]. That is why in 2009, Fernández de las Peñas et al. went further, observing that in TMDs, initial muscular pain might lead to a gradual sensitization of nociceptive pathways, eventually becoming chronic [9].

Furthermore, various studies have demonstrated that TMDs are related not only to masticatory muscle disorders, but also to some tongue muscle pathologies [16]. Corsalini et al. found that two-thirds of patients with glossodynia are also affected by a TMD [16]. Another example is Costen’s syndrome, in which tongue disturbances are a result of a TMD [17]. However, the relationship between motor and sensory tongue injuries in patients with TMD has been questioned by various authors [16,17,18]. Although there is a lot of evidence about the impact of TMDs on orofacial sensory-motor functions, there is little research that studies tongue function, specifically maximum tongue force in patients with chronic TMDs. Research has demonstrated that women with chronic TMDs show greater difficulty in chewing, greater frequency and predominance of unilateral chewing, and deterioration in the swallowing process [19]. Other authors demonstrated that these patients used to have pain and fatigue, while chewing with fatigue is related to chronic pain [20]. Moreover, it is known that tongue motor control is very important for some vital functions, such as breathing, phonation, and feeding [21]. The tongue is actively involved in swallowing and chewing, participating in the deglutition process, in which the anterior part of the tongue must push the bolus against the anterior hard palate, and then push it back against the soft palate [22]. Marim et al. showed that patients with chronic TMDs have lower tongue force in relation to the chewing and swallowing processes [23].

Also, the trigeminal cranial nerve is known to be responsible for sensory innervation and the hypoglossal nerve for motor innervation of the tongue. Based on this, Gelfand et al. found that afferent proprioceptive fibers of the trigeminal nerve lingual ramus are anastomosed with the hypoglossal nerve, then pass through the ansa cervicalis and continue to the ventral ramus of the C2 cervical nerve [18]. Thus, a compression of the C2 ventral ramus generates tongue ipsilateral numbness in neck-tongue syndrome because of an altered tongue proprioception [18,24]. They also reported other effects of altered tongue proprioception, showing difficulties recognizing the tongue position, which could result in dysarthria for some patients [18]. Other studies have observed relationships between tongue force and tongue position [25] and between tongue force exercises and subjective perception of fatigue [26].

Thus, the main objective of this study was to compare the maximum force of the tongue between women with chronic TMD and asymptomatic women. A secondary objective was to establish any relationship between the pain and fatigue and the maximum force developed in these women. We hypothesized that women with chronic TMD would have less tongue force and a higher subjective perception of pain and fatigue than the asymptomatic women.

## 2. Materials and Methods

### 2.1. Study Design

This study is a cross-sectional study. The research was approved 20 January 2018 by the ethical committee of the Hospital Universitario La Paz (PI-3077). Asymptomatic individuals and patients were recruited from the Centro Superior de Estudios Universitarios La Salle and the Hospital Universitario La Paz, according to the Strengthening the Reporting of Observational Studies in Epidemiology 2009 [27].

The research team was composed of a more than twenty-years-experienced maxillofacial surgeon of the Temporomandibular Joint Unit of the Hospital Universitario La Paz, three experienced physical therapists, and three assessors. The assessors were trained by a more than 15-years-experienced physical therapist for 180 min on how to conduct the procedure, except for the force measurement, which was performed by one of the physical therapists. The force variable was always executed by an experienced physical therapist.

### 2.2. Selection Criteria and Description of Participants

Women with chronic TMDs treated at the Hospital Universitario La Paz were recruited from February 2018 to September 2021 for the TMD group. The inclusion criteria were as follows: age between 18 and 65 years; female; previously diagnosed by an experienced maxillofacial surgeon from the Temporomandibular Joint Unit (Hospital Universitario La Paz); have a diagnosis based on the DC/TMD [5]; and have pain chronicity (>3 months continuously or >6 months intermittently), regardless if they were or not with TMD treatment.

Women from the local community of Madrid and the relatives or companions of the patients of the Hospital Universitario La Paz were recruited from February 2018 to April 2022 for the asymptomatic group. Participants were included if they met all the following criteria: age between 18 and 65 years; female; had not experienced any craniofacial/temporomandibular/neck pain; had no facial palsy caused by a primary muscle disorder; and had no significant history of chronic pain disorder.

The exclusion criteria were the same for both groups and avoided risk of bias: currently receiving physical therapy treatment for the neck or craniofacial region; a surgery or history of traumatic injuries of the neck/head/face/tongue/teeth/jaw; cancer or an active infection of the neck/head/mouth; rheumatic disorders; neurological disorders; and present a diagnosis of oral dysphagia. Each included participant signed an informed consent document and was allocated to the appropriate group: (a) the TMD group (TMD) or (b) the control group (CG).

### 2.3. Study Description

In this study, a non-probabilistic sample was used. After the participants were divided into the chronic TMD and CG groups, the procedure was the same for both: all participants had to be measured during one session, which was divided into two different parts. During the first part, physical variables, such as the intensity of perceived orofacial pain from now on and the intensity of perceived orofacial fatigue from now on, were measured. The second part consisted of the measurement of the maximum tongue force (MTF) by a smooth and rounded force sensor. Five different tongue movements were assessed with a well-validated instrument [28]. First, lip force was registered, then tongue force against the superior part of the hard palate was measured, followed by the tongue force against the jaw. Consecutively, the tongue force against the right and left buccinators was assessed. Finally, the session concluded by recording the intensity of perceived orofacial pain and the intensity of perceived orofacial fatigue after those tasks.

### 2.4. Outcome Measurements

Sociodemographic, somatosensory, and motor variables were collected for the study by various instruments and specialized registration tools and devices.

#### 2.4.1. Primary Outcome Measurements

Maximum tongue force (MTF) is the maximum force that the tongue can generate against a device. A validated prototype device was used to measure this variable [28]. The device was specifically created for this study. It consists of a base structure that disseminates the force made by the tongue in the appropriate direction to the sensor. The device uses force-sensitive resistor sensors, which transfer the data to the pertaining computer by an Arduino device. The data collected can be displayed in real time on the computer and stored in the system. Moreover, after storage, a processing system based on Java makes it possible to generate graphics through an interface. The unit of measure for this device is Newton (kg/F. 1 N = 0.101972 kgf). The MTF test includes 5 lingual movements: (1) Lips force (LF); (2) Palate force (PF)—with the device placed in the region immediately behind the upper incisors, the subject performs the strength in an anterior and cranial direction against the device; (3) Mandibular force (MF)—performed symmetrically, but in an anterior and flow direction against the device placed immediately behind the lower incisors; (4) Right cheek force (RCF)—with the device placed in the inner region at the right cheek, the subject exerts strength in a lateral direction by pressing the device; (5) Left cheek force (LCF)—with the device placed in the inner region at the left cheek, the subject exerts strength in a lateral direction against the device.

#### 2.4.2. Secondary Outcome Measurements

Intensity of perceived orofacial fatigue is the intensity of the fatigue that the patient subjectively experiences and recognizes as usual fatigue in the orofacial region. Fatigue was quantified through the numerical rating scale of fatigue. It consists of asking subjects about their level of fatigue on a scale from 0 through 10, in which 0 represents “no fatigue” and 10 represents “worst imaginable fatigue”. It has shown good reliability [29].

Intensity of perceived orofacial pain is the intensity of the pain that the patient subjectively experiences and recognizes as usual pain in the orofacial region. The “Numeric Pain Rating Scale” was used for its measurement, which is composed of 11 points ranging from 0 to 10, starting from the point of “no pain” represented at point 0 and up to the point of “the greatest pain you can imagine” represented at point 10. This scale has proven to be valid and reliable for measuring pain intensity (CHF = 0.95) [30], and its minimal detectable change in patients with chronic pain has been determined to be around a reduction of at least 2 points, or 30% [31,32,33].

#### 2.4.3. Procedure

**Figure 1:**
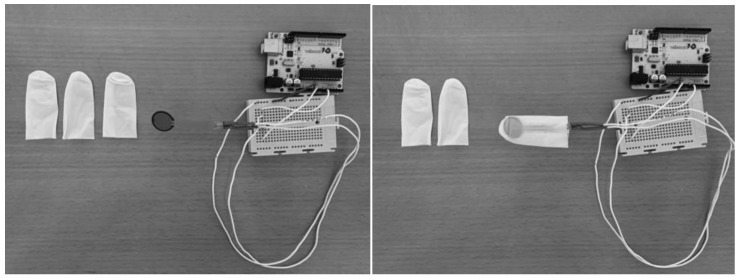
Prototype device.

**Figure 2:**
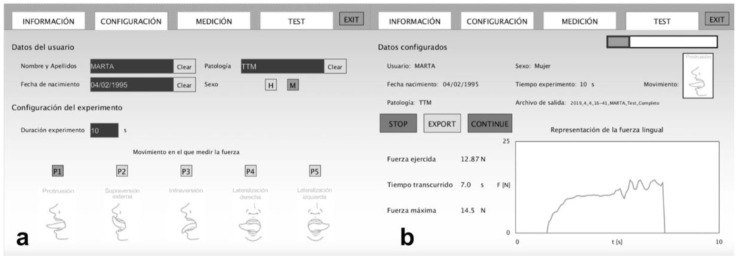
Spanish prototype device interface view. (**a**): Configuration screen view; (**b**): Measurement screen view.

**Figure 3:**
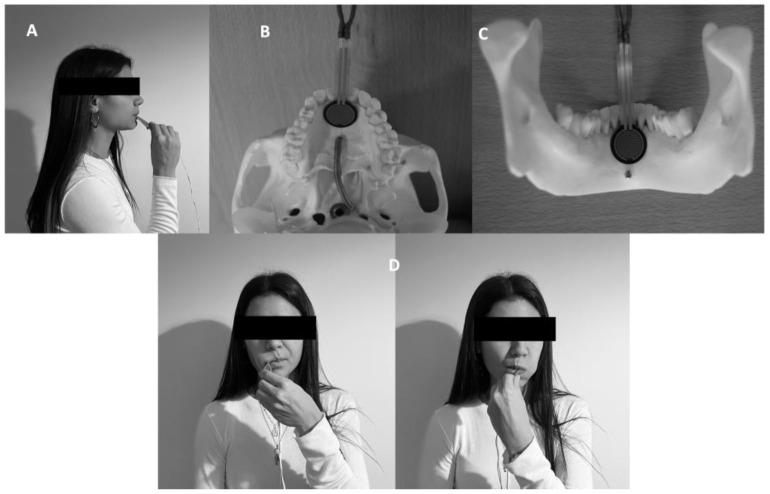
Tongue movements: (**A**) Force pressing lips together without teeth contact; (**B**) Force pressing tongue against the superior part of the hard palate; (**C**) Force pressing tongue against the jaw; (**D**) Force pressing tongue against the right and left buccinators, respectively.

First, an informed consent document was given to the participants; after reading and signing it, they provided sociodemographic data and answered various questions in relation to the inclusion and exclusion criteria. Then, participants were asked to sit in a chair with their feet on the floor, and each subject filled in a series of questions to collect clinical data and confirm that the inclusion and exclusion criteria were met. After that, the experienced physiotherapist confirmed the maxillofacial surgeon TMD diagnosis with the DC/TMD classification. Later, data were collected from the orofacial pain and the orofacial fatigue before the MTF test. The second part of the procedure began with measurement of the maximum tongue force. The participant sat in front of the device and put a single-use protection cover over the sensor like a sleeve (Figure 1). Before beginning the measurement, the assessor always checked the proper functioning of the system (Figure 2). Then, 5 various tongue movements were explained to the participant. Every movement needed to be maintained for 10 s, with verbal feedback from the assessor. The first movement was to generate force by pressing lips together without teeth contact. The tongue then had to press against the superior part of the hard palate, followed by a tongue thrust against the jaw. The final movements were to push the tongue against the right and left buccinators, consecutively. The movements are shown in Figure 3. Finally, the orofacial fatigue post and the orofacial pain post were measured by the numeric rating scale for fatigue and pain, respectively. All measurements were collected in a specific results document for each participant, which were stored by the principal investigator.

### 2.5. Bias

To avoid selection bias, inclusion and exclusion criteria were established, and subjects were pre-diagnosed for chronic TMD, according to ”Diagnostic Criteria for Temporomandibular Disorders” by the maxillofacial surgeon. To avoid reporting bias, all subjects received understandable information about the term “orofacial fatigue”. An investigator pre-demonstrated the MTF test, where the placement of the device in their mouth for each tongue movement was explained verbally and visually, providing subjects with pre-test familiarization with the device, as other authors proposed [34]. Subjects were not allowed to visualize the test results while exerting force, nor were they allowed to see the result, to avoid learning and influencing the results [35]. To avoid the MTF data being influenced by the age of the participants, the inclusion criteria was set to 70 years, taking into account that women over 70 years have been found to have significantly less maximum isometric tongue force [35].

### 2.6. Statistical Analysis

The Statistical Package for Social Sciences (SPSS 25, SPSS Inc., Chicago, IL, USA) software was used for the statistical analysis. For all the analyses, statistical significance was set at *p* < 0.05. Descriptive statistics were used to summarize the data for the continuous variables, which are presented as mean ± standard deviation (SD) and 95% confidence interval (CI), and categorical variables as an absolute number or relative frequency percentage. For all the variables, the Z-score was assumed to follow a normal distribution based on the central limit theorem because both groups had more than 30 subjects [36,37].

Comparisons between quantitative variables for independent samples were made using the T-Student test to analyze the continuous parametric data; the group factor was analyzed for the measurements. In addition, to quantifying the size of the difference between both groups, the effect sizes (d) were calculated according to Cohen’s method, in which the magnitude of the effect was classified as small (0.20–0.49), medium (0.50–0.79), or large (0.8) [38]. Finally, to test the relationship between pain and fatigue outcomes with tongue force variables, Pearson’s correlations coefficient (r) was calculated separately for each group. This coefficient (r) was classified as weak (0–0.3), moderate (0.3–0.5), or strong (0.5–1).

## 3. Results

A total of 67 women, 36 with chronic TMD and 31 asymptomatic, between the ages of 32 and 65 (46.67 ± 10.80 and 49.06 ± 11.19, respectively), were included in the study and were categorized into 2 groups: chronic TMD or CG. Baseline characteristics of the age of the sample showed no significant differences between the two groups (*p* = 0.38). No patients dropped out during the study, and no adverse events occurred during the test.

### 3.1. Maximum Tongue Force

The analysis showed significant differences (*p* < 0.05) for this variable between groups. There are significant differences for all the movements, except for LF (*p* = 0.07). The most significant differences were obtained for the PF (*p* < 0.01). Descriptive data for the five force measurements are presented in Table 1.

### 3.2. Intensity of Perceived Orofacial Fatigue

The statistics revealed significant differences between the TMD and CG for orofacial fatigue pre (*p* < 0.01) and orofacial fatigue post (*p* < 0.01). Results are shown in Table 2.

### 3.3. Correlations

Pearson’s correlation coefficient proved the relationship between some of the studied measurements. The data analysis revealed small correlations (0.1–0.3) between MTF and orofacial pain and orofacial fatigue. Correlations data are presented in Table 3.

## 4. Discussion

In the present study, we compared the maximum tongue force between women with chronic temporomandibular disorders and asymptomatic women. Likewise, we studied the intensity of perceived orofacial fatigue between groups and the correlations between maximum tongue force and the intensity of perceived orofacial pain and the intensity of perceived orofacial fatigue. The main results that can be drawn from this study are as follows: there are differences in the maximum tongue force and the intensity of perceived orofacial fatigue between groups, but there are no correlations between maximum tongue force and the intensity of perceived orofacial pain and the intensity of perceived of orofacial fatigue.

In this study, the chronic TMD group and the CG revealed a significant difference between groups for the MTF. The TMDs group obtained lower MTF than the CG in all the movements, except for the LF, which has a strong clinical implication and introduces new approaches. The fact is that few studies have been published to date in terms of the relationship between TMDs and MTF. Only two studies were found that specifically measured MTF in TMDs, but they did not evaluate all the movements. Due to the lack of literature, we can only compare our results with studies that evaluated MTF in one or two of these movements. According to this, Rosa et al. studied tongue elevation and protrusion force in patients with TMDs [39]. Likewise, Marim et al. only evaluated tongue protrusion force in these types of patients [23]. As in our study, these research studies reported reduced tongue force in chronic TMDs patients. Additionally, they showed that chronic TMDs patients with less tongue force had more difficulty during swallowing and chewing. These results lead them to consider the importance of evaluating impairments in orofacial muscles other than jaw elevator muscles for chronic TMDs patients. This is in concordance with our results for the MTF in chronic TMDs, compared to asymptomatic subjects. Similar research has shown more functional disorders in the masticatory muscles during chewing. Weber et al. did not measure MTF, but they found a correlation between abnormal tongue and lip behaviors in patients with TMDs, supporting the influence of the tongue in these patients [40]. Moreover, we found a study that demonstrates the importance and implications of training the tongue force in rats. Klezien et al. performed a study in which rats were divided into three groups: tongue exercises, treadmill exercises, and no exercises. After 8 weeks with 5 days per week of exercises, the authors found that the tongue exercise group had more contractile properties in the tongue, compared with the other groups [41].

Further, it is important to highlight that among the different tongue movements, the most studied is the one where the force is exerted against the anterior hard palate in its anterior portion [42], referred to in our study as PF. According to Adams et al., the mean values for this measure of PF in healthy adults are between 56.50–73.33 kPa (men and women) [42] and between 41–69.80 kPa, according to the most current systematic review [43]. In addition, specifically in healthy adult women, values between 56.50–61.27 kPa have been found [42]. Based on that and comparting with the data obtained in our study for PF in the TMD group (6.36 N = 50.21 kPa), women with chronic TMDs present below-normal PF values vs. healthy adult women. The TMD group presented a PF deficit of about 6.29 kPa. This finding is relevant because it has been described that a force against the anterior hard palate must be exerted in the swallowing process [21], more significantly when swallowing liquids compared to solids [43]. Therefore, women with chronic TMDs may show lower PF values in association with swallowing disorders. Fassicollo et al. agreed with this hypothesis and found that patients with chronic TMDs need more time to perform fluid and saliva swallowing, which could be due to weak tongue function [44]. We hypothesize that the swallowing dysfunction observed in patients with chronic TMDs may be due to a significant decrease in the PF.

Although many studies have measured intra- and extra- oral tongue force protrusion, only one has studied it in patients with chronic TMDs. This study found significantly lower values in the patients group, compared to the control group [23], suggesting that it is a relevant movement to measure for the study of these patients. It should be noted that our study has proposed a new way to measure this movement, exerting the force against the internal surface of the jaw, with the tongue in a first retraction position. Doing this intraoral movement against a rigid surface could be easier and more comfortable for patients, allowing them to exert the greatest force. This is in line with a study carried out on healthy subjects, which showed that the highest force values were developed when the tongue protrusion was performed in this position [45]. Moreover, we obtained tongue force protrusion values that complement the mean values found for this movement by Marim et al. [23].

For the lateral tongue movements, we have literature that specifically analyzed it, but there is no clear consensus on how to perform the measurements. The studies measured lateral tongue force using devices that guarantee a fixed lateral resistance, against which the tongue exerts an isometric contraction. However, we propose to carry out the measurement by pressing the device laterally with the tongue against the cheeks. Even though the elasticity of the cheeks could influence the tongue force and could be a limitation to objectively measuring this movement, we consider that this position will make it easier and more comfortable for the participants and avoid the use of supplementary material for the device, altering the ability of the subjects to perform the test after introducing it into the mouth. This is the first study that has measured the lateral tongue force in this position, and the data obtained show significant differences between these variables. This could suggest the need to analyze these differences with the aim of specifically studying the presence of asymmetry of lateral tongue force in TMDs patients.

Otherwise, we obtained differences between groups for the orofacial fatigue and the orofacial fatigue posttest. Our results agree with previous studies, such as that by Kletzien et al., which observed that tongue exercises were related to some increase in fatigue [41]. Eisenlohr-Moul et al. found that the lack of pain acceptance and tolerance in patients with chronic TMDs with longer duration of symptoms increased the orofacial fatigue [46]. Symptoms of TMDs could be understood as a complex response to the central sensitization caused by pain chronification [47]. In fact, these findings could explain the results that we obtained for the orofacial fatigue in the TMD group.

In contrast, the present study showed small correlations between MTF and orofacial pain and orofacial fatigue. This is contrary to the literature that obtained medium and large correlations between the muscle activation and the orofacial fatigue and orofacial pain in patients with myofascial pain-dysfunction syndrome. Gay T et al. demonstrated that these types of patients had less activation of the masseter muscles and fatigue symptoms than the asymptomatic subjects [48]. Likewise, a systematic review of the literature analyzed the orofacial fatigue and the duration of symptoms of chronicity in patients with rheumatic disease. They showed that the longer the pain experience, the more fatigue perceived, and the greater the disease activity, the greater the fatigue experienced [49]. In line with this, a study by Wozniak et al. analyzed the correlation between patients with TMD and asymptomatic individuals for masticatory muscle fatigue. They concluded that the orofacial fatigue in those muscles was related to TMD symptom intensity [50]. It is known that fatigue is the result of a decrease in the ability to generate the force of a muscle, which results from recent activity, physiologic reaction, or pathologic condition [51]. In this way, the evidence is used to show important correlations between muscle force and orofacial fatigue. However, the literature has proved that muscle fatigue is influenced by age, gender, health status, physical condition, and psychosocial conditions, among others [52]. Moreover, it has been demonstrated that after the fatigue of a single muscle, there are compensating mechanisms and control strategies between synergistic muscles in order to solve the problem. Some studies revealed that after the fatigue of a muscle, the voluntary muscle activity of the synergistic muscles increase significantly, solving the activity with similar results [53]. This is the reason why we hypothesized that our study would not obtain important correlations for these variables. Some external conditions or compensating mechanisms could be involved in our sample.

According to all the information above, the study reveals some changes in the MTF and the orofacial fatigue of patients with TMDs that should be considered for further investigation because this can have important clinical implications, not only for the assessment process, but for the clinical and home treatment of these patients, too. This study reveals a need to open a new line of research to implement and strengthen a program for the tongue in order to reduce craniofacial pain and fatigue. Future studies, in which we can establish normal values in this population using the new tongue force device that has been used in this study, should be conducted. Moreover, studies comparing treatments that include tongue force training vs. treatments that only train motor control exercises in TMDs patients should be developed.

### Limitations

This study has some limitations. First, the instrument does not have a mandibular fixation while the patient develops tongue force. This could prevent compensatory movements when the deficit of tongue force leads to the use of mandibular strength [54]. Second, tongue force against the right and left buccinators could be a limitation because the force is made against two soft-tissue surfaces. Third, we must highlight that we used a non-probabilistic sample and that implies less precision in the represented population. This type of sample does not allow generalization regarding the whole population. Moreover, we have to highlight that there is heterogeneity in the TMDs group because we did not compare and analyze between the articular, muscular, or mixed TMD conditions; future studies should distinguish between them. Likewise, we did not confirm that the pain was related to the TMD. Finally, the diagnosis was made by a maxillofacial surgeon; future studies may include a classification or function questionnaire that may help to provide more data. Future research should attempt to address these limitations.

## 5. Conclusions

In conclusion, the results showed significant differences in the MTF when comparing women with chronic TMDs and asymptomatic women. The chronic TMDs group revealed less MTF than the CG after developing all tongue movements, except the LF. Moreover, the data obtained a higher weakness in the PF, compared to the other movements, due to it being the most developed one during vital functions. Likewise, the orofacial fatigue revealed significant differences for both groups. However, this study reveals small correlations between the orofacial pain and orofacial fatigue and the MTF.

## Figures and Tables

**Table 1:** Values for maximum tongue force (Newton).

	TMD Group (Mean ± Standard Deviation)	Control Group (Mean ± Standard Deviation)	*p* Value	Mean Difference (CI)	Effect Size (d)
**Lips Force**	2.52 ± 2.69	3.61 ± 2.03	0.07	−1.09 (−2.27 to 0.08)	−0.45
**Palate Force**	6.39 ± 7.07	14.38 ± 9.80	<0.01 *	−7.99 (−12.24 to −3.74)	−0.95
**Mandibular Force**	5.54 ± 7.26	9.53 ± 6.33	0.01 *	−3.98 (−7.30 to 0.67)	−0.58
**Right Cheek Force**	1.79 ± 2.06	3.89 ± 3.34	<0.01 *	−2.1 (−3.49 to −0.71)	−0.77
**Left Cheek Force**	1.98 ± 1.79	3.92 ± 3.00	<0.01 *	−1.94 (−3.18 to −0.7)	−0.80

TMD: Temporomandibular Disorders; Lips Force: lip to lip force; Palate Force: tongue against superior part of the hard palate; Mandibular Force: tongue against the jaw; Right Cheek Force: tongue force against the right buccinator; Left Cheek Force: tongue force against the left buccinator; CI: Confidence Interval; *p* value and Cohen effect size (d); *: *p* < 0.01.

**Table 2:** Values for Intensity of Perceived Orofacial Fatigue.

	TMD Group (Mean ± Standard Deviation)	Control Group (Mean ± Standard Deviation)	*p* Value	Mean Difference (CI)	Effect Size (d)
**Orofacial Fatigue pre test**	3.31 ± 3.16	0.06 ± 0.36	<0.00	3.24 (2.16 to 4.31)	1.39
**Orofacial Fatigue post test**	6.04 ± 2.71	2.16 ± 2.25	<0.00	3.88 (2.65 to 5.1)	1.55

TMD: Temporomandibular Disorders; Orofacial Fatigue pre test: the intensity of orofacial fatigue before developing the 5 tongue force movements; Orofacial Fatigue post test: the intensity of orofacial fatigue after developing the 5 tongue force movements; CI: Confidence Interval; *p* value and effect size (d).

**Table 3:** Pearson´s correlations.

	Lips Force	Palate Force	Mandibular Force	Right Cheek Force	Left Cheek Force
**Intensity of Perceived Orofacial Pain**	−0.28 *	−0.29 *	−0.25 *	−0.20	−0.13
**Intensity of Perceived Orofacial Fatigue**	−0.22	−0.22	−0.20	−0.21	0.12

* *p* < 0.05.

## Data Availability

Not applicable.
